# SUMOylation in *Drosophila* Development

**DOI:** 10.3390/biom2030331

**Published:** 2012-07-25

**Authors:** Matthew Smith, Wiam Turki-Judeh, Albert J. Courey

**Affiliations:** Department of Chemistry & Biochemistry and Molecular Biology Institute, University of California-Los Angeles, 607 Charles E. Young Drive East, Los Angeles, CA 90095-1569, USA; Emails: msmith@chem.ucla.edu (M.S.); wiamtj@ucla.edu (W.T.-J.)

**Keywords:** SUMO, Ubc9, chromatin, pattern formation, wing morphogenesis, Ras signaling, metamorphosis, innate immunity

## Abstract

Small ubiquitin-related modifier (SUMO), an ~90 amino acid ubiquitin-like protein, is highly conserved throughout the eukaryotic domain. Like ubiquitin, SUMO is covalently attached to lysine side chains in a large number of target proteins. In contrast to ubiquitin, SUMO does not have a direct role in targeting proteins for proteasomal degradation. However, like ubiquitin, SUMO does modulate protein function in a variety of other ways. This includes effects on protein conformation, subcellular localization, and protein–protein interactions. Significant insight into the *in vivo* role of SUMOylation has been provided by studies in *Drosophila* that combine genetic manipulation, proteomic, and biochemical analysis. Such studies have revealed that the SUMO conjugation pathway regulates a wide variety of critical cellular and developmental processes, including chromatin/chromosome function, eggshell patterning, embryonic pattern formation, metamorphosis, larval and pupal development, neurogenesis, development of the innate immune system, and apoptosis. This review discusses our current understanding of the diverse roles for SUMO in *Drosophila* development.

## 1. The SUMO Conjugation Pathway in *Drosophila*

As its name implies, small ubiquitin-related modifier (SUMO) is a Ubiquitin-Like protein (UbL) with sequence and structural similarity to ubiquitin [1]. While the human genome encodes at least four SUMO family proteins [2,3], the *Drosophila* genome encodes one such protein [4] which is more similar to human SUMO-2/3/4 than it is to human SUMO-1. Although the *Drosophila* gene encoding SUMO was originally termed *smt3* by analogy with its yeast counterpart, we will use the more familiar “*SUMO*” (italicized for the gene name, but not for the protein) throughout this review. Like ubiquitin, SUMO becomes covalently attached to lysine side chains in a variety of target proteins in a process termed SUMOylation. It is synthesized as an inactive precursor that must undergo maturation before it can proceed through the enzymatic steps required for conjugation to target proteins. Maturation of SUMO is accomplished by the activity of a ubiquitin-like protease (Ulp), which removes a C-terminal extension from the immature protein exposing a Gly-Gly motif at the C-terminus. The Ulp family is similar to the sentrin/SUMO-specific protease (SENP) family in humans. In *Drosophila* SUMO, this C-terminal extension is just two residues in length [5].

Once processed, the mature SUMO is ready to enter the three-step conjugation pathway. In the initial step, which is coupled to the hydrolysis of ATP to AMP and pyrophosphate, the C-terminal carboxyl group of SUMO forms a thioester bond with an active site cysteine of the heterodimeric SUMO activating enzyme SAE1/SAE2. This thioester linkage is then transferred to an active site cysteine within the conjugating enzyme Ubc9 (which is encoded by the *Drosophila* gene *lesswright* (*lwr*)) [6]. Ubc9 is capable of recognizing the substrate and catalyzes the formation of an isopeptide bond between the C-terminus of SUMO and an ε-amino group of a lysine residue within the substrate. The target lysine residues usually fall within a SUMO conjugation consensus motif Ψ KxE (where Ψ is a large hydrophobic residue and x is any amino acid) [7]. Isopeptide bond formation can be facilitated by the activity of SUMO E3 ligases that bind both Ubc9 and the target, increasing the rate of SUMO conjugation to a specific target or, in some cases, promote poly-SUMO chain formation [8]. In *Drosophila*, it is likely that the protein inhibitor of activated STAT (PIAS) family protein suppressor of variegation 2-10 (Su(var)2–10) functions as a SUMO E3 ligase [9,10].

SUMO conjugation is readily reversed by the action of the Ulps, which function as deconjugases in addition to maturases. Thus, for many targets, the conjugate appears to be transient and it is possible that the transient nature of the modification is an important feature in SUMO function [5]. The Ulps are often found in specific subcellular locales and their ability to mediate deconjugation in a cell-compartment specific manner may play an important role in controlling SUMO pathway function. The *Drosophila* genome encodes at least two such deconjugases, termed Ulp1 and Ulp2. The first of these, Ulp1, has been extensively characterized [5]. It is primarily associated with the nucleoplasmic face of the nuclear pore complex where it may deconjugate proteins as they exit the nucleus thus serving as a molecular switch to control the biochemical properties of a protein as a function of its subcellular location.

## 2. Roles for SUMO in Chromosome/Chromatin Function

### 2.1. Roles in the Meiotic and Mitotic Chromosome Cycles

SUMO may have roles in both meiotic and mitotic chromosome function. The earliest evidence for a role in meiosis was provided by the discovery that mutations in the gene encoding the SUMO conjugating enzyme Ubc9 can suppress the *no distributivedisjunction* (*nod*) meiotic nondisjunction phenotype. It appears that Ubc9 promotes the dissociation of homologous heterochromatic regions at the end of meiotic prophase I [11]. Evidence for a role of the SUMO pathway in coordinating the mitotic chromosome cycle comes from the discovery of chromosomal hypercondensation and aberrant segregation defects observed in *SUMO* loss-of-function embryos. SUMO is required for progression through the cell cycle and, consistent with this observation, numerous cell cycle regulators have been identified as SUMO conjugation targets [12].

### 2.2. Roles in Insulator Function

Further evidence for a role of SUMO in chromatin function is provided by the finding that SUMO contributes to the ability of the gypsy transposon to insulate chromosomal domains. Two protein components of the *gypsy* chromatin insulator complex, Mod(mdg4)2.2 and CP190, can be modified by SUMO *invitro* and *invivo*. SUMOylation of these insulator components may antagonize insulator activity as disruption of the SUMO pathway can increase the enhancer blocking activity of the insulator. SUMOylation does not affect the ability of CP190 and Mod(mdg4) to bind chromatin, but rather appears to regulate the stability of gypsy insulator complexes. For example, Ubc9 overexpression leads to disintegration of such complexes, whereas reduced levels of SUMOylation partially restore insulator body formation lost due to the absence of Mod(mdg4) [13]. SUMOylation of these insulator body components is apparently regulated by dTopors, a dual ubiquitin/SUMO ligase, which could stimulate insulator body function by interfering with SUMOylation of CP190 and Mod(mdg4). However, further study is required as published data regarding the role of SUMO and Topors in *gypsy* insulator function are inconsistent [13,14,15,16].

### 2.3. Connections to Heterochromatic Silencing

Links between SUMO and chromatin function are also suggested by the multiple connections between the SUMO pathway and position effect variegation (PEV). PEV arises from the establishment of mitotically stable transcriptionally silent heterochromatic domains [17]. Suppressors of PEV encode proteins that favor heterochromatic silencing, and one such gene, Su(var)2-10, encodes the *Drosophila* orthologue of the human SUMO E3 ligase, PIAS, [18]. Furthermore, the conjugation of SUMO to lysine 839 in another such gene, Su(var)3-7 is required to target it to heterochromatic regions and promote PEV [19].

### 2.4. Links to Polycomb Group Function

SUMO may also be required for the function of the Polycomb group (PcG). This set of gene products functions to establish and maintain an epigenetically stable silent transcriptional state that has some similarity to the heterochromatic state [20]. In *Drosophila* development, the PcG serves to maintain the silent transcriptional state of the homeotic complex (*H*ox) genes, such as *Ultrabithorax* (*Ubx*), throughout the latter stages of embryogenesis and throughout larval and pupal development. The PcG includes two protein complexes termed Polycomb Repressive Complexes 1 and 2 (PRC1 and PRC2) that interact with cis-regulatory elements termed Polycomb Response Elements (PREs) to maintain a transcriptionally silent state.

Evidence that SUMO is required for PcG function came from the discovery that Sex comb on midleg (Scm), a substochiometric but nonetheless essential component of PRC1, is efficiently sumoylated in vivo. In S2 cells, Scm SUMOylation interferes with its association with the major Ubx PRE. Thus, depletion of SUMO by RNAi or mutation of the three SUMO-acceptor sites in Scm lead to increased association of Scm with the PRE and increased repression of *Ubx*, whereas fusion of SUMO to the N terminal region of Scm interfered with the recruitment of Scm to the PRE [21].

**Figure 1:**
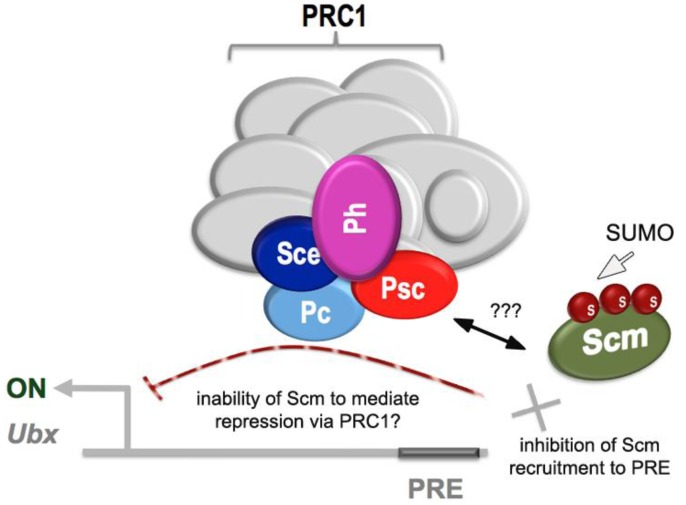
Role of SUMOylation in Sex comb on midleg (Scm) protein function.SUMOylation (S) of Scm blocks its recruitment to the *Ultrabithorax* (Ubx) Polycomb Response Element (PRE) and thus inhibits Polycomb Repressive Complexes 1 (PRC1)-mediated repression of Ubx. SUMOylation of Scm could also interfere with the binding of PRC1 to Scm, but this idea has not been directly tested.

Evidence that SUMO function in the PcG pathway is biologically relevant comes from the observation that SUMO has a role in determining the identity of the third thoracic segment in the adult fly. Classic studies on the function of *Ubx* as a selector gene showed that its function in the third thoracic segment from which the halteres arise is required for the identity of this segment [22]. When Ubx is removed from the third thoracic segment, the resulting homeotic transformation converts this segment into a second thoracic segment with the accompanying transformation of the halteres into wings. As mentioned above, cell culture studies show that SUMO functions via Scm to prevent PcG hyperactivity and thus over-repression of *Ubx*. Consistent with this observation, when SUMO is depleted from developing halteres via clonal RNAi in the haltere disc, the result is a partial transformation of the haltere to a wing presumably due to inappropriate repression of Ubx by the PcG [21]. Thus, SUMO may negatively regulate Scm function by inhibiting its recruitment to the Ubx major PRE (Figure 1).

SUMO may also modulate the function of other PcG proteins. For example, Pleiohomeotic, a DNA binding transcription factor with a primary role in PRE recognition, is a SUMO conjugation target [12]. Furthermore, the human PcG protein Pc2 acts as a SUMO E3 ligase, and recruits Ubc9 and the transcriptional corepressor CtBP into subnuclear compartments termed Polycomb bodies, where SUMOylation of CtBP may occur [23].

## 3. A Role for SUMO in Eggshell Patterning

A role for SUMOylation in regulating eggshell patterning was first suggested by the observation that *SUMO* hypomorphic mutations act as enhancers of the hypomorphic *Ras1* ventralized eggshell phenotype [24]. During oogenesis, the Ras signaling cascade is activated in the follicle cells that surround the developing oocyte by the transmembrane receptor tyrosine kinase (RTK) epidermal growth factor receptor (EGFR) to pattern the dorsoventral axis of the eggshell, which is synthesized by the follicle cells [25]. Since Ras signaling is required for specification of the dorsal pattern elements in the eggshell including two processes termed the dorsal appendages, mutations in components of the Ras signaling cascade result in ventralized eggshells as manifested by dorsal appendage defects. SUMO mutations enhanced this phenotype. Furthermore, reduced SUMO levels suppress the eggshell dorsalization that results from expression of an activated form of EGFR suggesting that SUMO acts downstream of EGFR in the follicle cells [24].

Further support for the conclusion that SUMO acts downstream of EGFR was provided by experiments examining activation of the Ras signaling pathway components mitogen activated protein kinase (MAPK) and MAPK/Erk kinase (MEK) in EGFR-expressing S2R+ cells. Activation of the Ras pathway with the EGFR ligand Spitz or the insulin-like receptor ligand insulin induces phosphorylation of both MAPK and MEK, and this phosphorylation was significantly attenuated in cells depleted of SUMO by RNAi. In contrast, depletion of SUMO had no effect on MAPK activation by a constitutively active form of Ras1 suggesting that SUMO is required for Ras activation [12].

The possibility that SUMO modulates Ras1 function directly is supported by two observations. First, Ras1 can be efficiently sumoylated at multiple lysine residues present within its C-terminal hypervariable region. Second, Ras1 was identified as a SUMO-associated protein in a proteomic screen to identify novel SUMOylation targets in the early embryo. This screen also identified many other proteins involved in Ras signaling as either SUMOylation substrates or SUMO-interacting proteins suggesting that eggshell patterning is regulated at multiple levels by SUMO-modulation of Ras pathway activity [12].

## 4. Embryonic Patterning

### 4.1. Anteroposterior Patterning

Maternally deposited SUMO and SUMO conjugation pathway components (Ubc9, SAE1, SAE2, and Ulp1) are present at high concentrations in the early *Drosophila* embryo [6,26,27]. The first evidence for a role of SUMOylation in embryonic pattern formation came from an analysis of the embryonic phenotype resulting from loss-of-function mutations in the gene encoding Ubc9. Such mutations lead to a loss of anterior segments, a phenotype that resembles those that result from mutations in *hunchback* (*hb*). Reduction of Ubc9 levels in the early embryo produce defects that range from a missing first thoracic segment to the absence of all segments from the first thoracic to the fifth abdominal segment [28]. Consistent with this phenotype, a proteomic screen showed that both Hb and its positive regulator Bicoid are SUMOylation targets in the early embryo and that mutations in the gene encoding SUMO produce a range of defects that include anteroposterior defects [12]. Interestingly, SUMO appears to have opposing, direct and indirect effects on Bicoid function. Experiments looking at embryos with reduced Ubc9 levels suggest that SUMOylation is required for Bcd function [28], while direct modification of Bcd by SUMO appears to negatively regulate Bcd-dependent transcriptional activation [29].

### 4.2. Dorsoventral Patterning

Mutations in the gene encoding SUMO also lead to dorsoventral patterning defects [12], an observation that is consistent with the finding that the Rel family morphogen Dorsal is sumoylated in the early embryo [12]. Studies of Dorsal SUMOylation in cultured cells suggest that SUMOylation stimulates Dorsal-dependent transcription [30,31] although the mechanism behind this stimulation is not clear.

An additional and better understood role for the SUMO pathway in directing dorsoventral pattern formation is in Decapentaplegic (Dpp) signaling [32]. Dpp is a TGF-β family ligand that is distributed in a concentration gradient in the extraembyonic space on the dorsal side of the early embryo. Dpp signals through transmembrane receptors to trigger the activation of target genes by SMAD family transcription factors, including the *Drosophila* Mad and Medea (Med) proteins. Specifically, the Dpp signal leads to the phosphorylation of Mad, which then enters the nucleus and activates Dpp target genes as a heterotrimer with Med [33,34].

The SUMO pathway may regulate patterning by restricting the range of action of the Dpp morphogen as suggested by experiments in which reduced SUMO pathway activity was found to result in expanded expression of Dpp targets. In accord with this finding, a triple mutant form of Med lacking all three potential SUMO acceptor sites increased Med transcriptional activity, leading to expanded expression of Dpp targets. Overexpression of a Med-SUMO fusion protein led to retraction of the gene expression pattern of Dpp target genes similar to what is observed in *dpp* heterozygous embryos. This suggests that Med SUMOylation negatively regulates Dpp target genes [32] and is in agreement with findings that demonstrate reduced Smad4 transcriptional activity when this mammalian Med homologue is sumoylated [35].

While it is not clear how Med SUMOylation reduces its activity, an effect of SUMOylation on Med mobility may play a role. This is suggested by experiments in which nuclei containing a GFP-Med fusion protein were photobleached followed by determination of the rate of recovery of nuclear fluorescence due to import of unbleached GFP-Med from the cytoplasm into the nucleus. Mutation of the three SUMO acceptor lysines in Med greatly reduced the rate of fluorescence recovery suggesting that SUMOylation of Med may increase its mobility [32].

**Figure 2:**
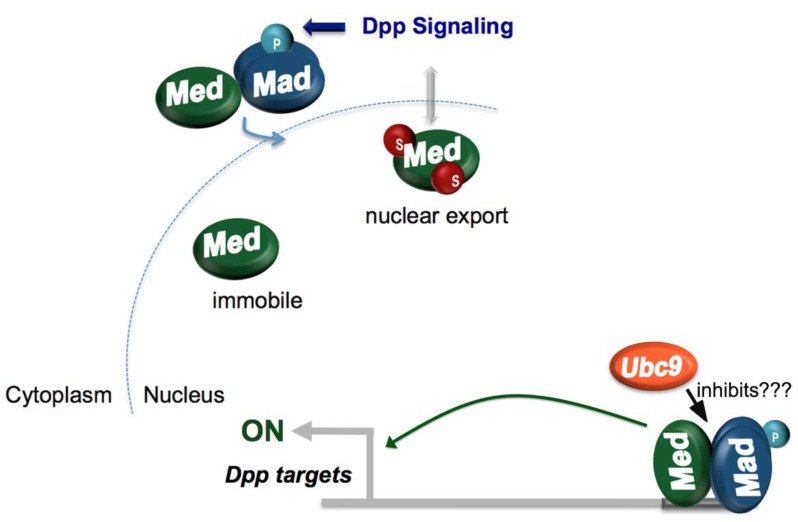
Med SUMOylation limits Dpp signaling by stimulating Med nuclear export. Decapentaplegic (Dpp) signaling enhances nuclear import of Med through an interaction with phospho-Mad (pMad). Once in the nucleus, Med is sumoylated. This increases Med mobility and may thus permit Med nuclear export, thereby limiting the time of Med residence in the nucleus. Since less sumoylated Med is detected in the presence of the Dpp signal, it is possible that phosphorylated Mad decreases the rate of Med SUMOylation perhaps by interfering with the Ubc9-Med interaction.

One possible model to explain the link between SUMOylation, Med mobility, and Dpp signaling is the following: First, it is known that Dpp signaling leads to Mad phosphorylation and that the resulting phospho-Mad then enters the nucleus bringing Med with it [33]. Once in the nucleus, Med would encounter the SUMOylation machinery (which is almost exclusively nuclear [5,26,27]) and become sumoylated. The increased mobility of sumoylated Med would then facilitate its export from the nucleus (Figure 2), explaining the broader nuclear distribution of Med that results from disruption of the SUMO pathway. Since the SUMO deconjugating enzyme Ulp1 resides in the nuclear pore complex, it is possible that Med would be de-sumoylated on its way out of the nucleus decreasing its mobility and thus decreasing the rate of Med re-import. This cyclic conjugation and deconjugation of Med during nucleocytoplasmic shuttling could thus render Dpp signaling self-limiting by ensuring that Med only enters the nucleus transiently after a Dpp signal is received, thereby preventing the inappropriate activation of Dpp target genes after signaling stops.

## 5. Wing Morphogenesis

### 5.1. Regulation of Vestigial Function by SUMO

SUMO appears to have multiple roles in wing morphogenesis. For example, SUMO modulates the function of the wing selector gene *vestigial* (*vg*). This gene, which encodes a transcriptional coactivator, is essential for wing formation and, furthermore, its misexpression can lead to ectopic wing formation. Mutations in *SUMO*, *Ubc9*, or *Su(var)2-10* all enhance the wing notching phenotype that results from reduced function of *vestigial* (*vg*). In addition, *SUMO* overexpression enhances the ectopic wing outgrowth phenotype that results from misexpression of *vg* in the eye imaginal disc [36].

### 5.2. Regulation of Sal and Salr by SUMO

A further role for SUMO in wing development is demonstrated by a study examining interactions between the SUMO pathway and the *Drosophila* Spalt-like (Sall) family proteins Spalt (Sal) and Spalt-related (Salr). These zinc finger transcription factors control growth and wing venation in the central part of the wing during larval and pupal development [37]. A vertebrate member of this family is a known SUMOylation target although the functional significance of its SUMOylation is not known [38].

Both loss and gain of function analyses were employed to determine if the SUMO pathway is required for the function of the *Drosophila* Sall proteins in the wing. Flies heterozygous for a deficiency that removes both *sal* and *salr* exhibited a modest decrease in wing size, an effect that was significantly increased upon removal of one copy of the gene encoding SUMO or the gene encoding Ubc9, implying a synergistic interaction between Sall proteins and the SUMO pathway. In addition, flies doubly heterozygous for the deficiency and the gene encoding SUMO exhibited ectopic vein material, a phenotype not observed in either single heterozygote [39].

Both Sal and Salr were found to contain two evolutionarily conserved potential SUMOylation sites and mutagenesis of critical aspartate or glutamate residues downstream of each SUMO acceptor lysine was found to significantly reduce SUMOylation [39]. Phenotypes due to overexpression of wild-type and mutant forms of Sall proteins were then assessed by examining wing size, wing venation, and the expression of *knirps*, a known target of Sall family proteins in the wing. Induction of ectopic wing vein formation and broadening of the *knirps* expression domain by Sal overexpression is partially suppressed by mutation of the SUMO acceptor sites in Sal. In contrast induction of ectopic wing vein formation and broadening of the *knirps* expression domain is enhanced by mutation of the SUMO acceptor sites in Salr. Thus, SUMO appears to affect Sal and Salr in contrasting ways.

To determine how SUMOylation might alter Sall protein functions, the effects of the SUMO on Sall protein subnuclear localization in S2 cells was assessed [39]. Overexpressed Sal and Salr both localized to punctate nuclear bodies. Co-overexpression of SUMO, presumably leading to increased SUMOylation, blocked punctate body formation in the case of Sal, but led to the formation of large nuclear aggregates in the case of Salr. The effects of SUMO co-overexpression were not observed when SUMOylation deficient mutants of Sal and Salr were employed in place of the wild-type Sall proteins. It thus appears that SUMO may differentially regulate the two *Drosophila* Sall proteins by differential affects on their subnuclear localization. Consistent with this view, SUMOylation differentially regulates Sal and Salr function in cell culture transfection assays [40]. 

### 5.3. Regulation of Homeodomain interacting protein kinase (Hipk) by SUMO

SUMO-mediated regulation of wing development via the modulation of protein subcellular localization may be a common theme in wing morphogenesis. In addition to the effect of SUMO on the Sall protein localization discussed above, SUMO also appears to alter the subcellular localization of the homeodomain interacting protein kinase (Hipk), thereby influencing c-Jun N-terminal kinase (JNK) signaling during wing development. JNK is a member of the MAP kinase family with multiple roles in development. Studies of vertebrate JNK signaling suggest that it might be regulated by Hipk family kinases [41,42], although the mechanistic basis for this regulation is not understood.

**Figure 3:**
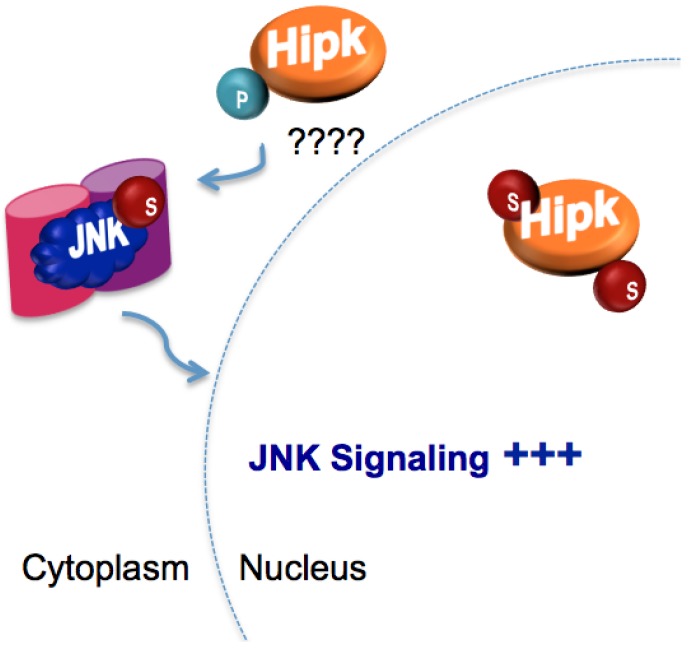
Homeodomain interacting protein kinase (Hipk) SUMOylation may down-regulate c-Jun N-terminal kinase (JNK) signaling. Phosphorylation of JNK in the cytoplasm by Hipk may stimulate JNK pathway activity. SUMOylation of Hipk may interfere with JNK signaling by sequestering Hipk in the nucleus.

SUMO depletion in the wing disc by RNAi leads to a marked increase in the number of apoptotic cells, a phenotype that was significantly reduced when JNK activity was reduced either by RNAi against JNK or through the use of a dominant negative JNK allele. In support of the hypothesis that SUMO depletion leads to apoptosis via increased JNK signaling, depletion of SUMO in the wing disc caused ectopic expression of downstream targets of JNK signaling. The upregulation of JNK signaling in SUMO-depleted wing discs required the action of Hipk. Sumoylated Hipk is found in the nucleus and reducing SUMO levels results in translocation of Hipk from the nucleus to the cytoplasm. These findings suggest that SUMOylation of Hipk serves to sequester it in the nucleus, and loss of Hipk SUMOylation allows it to be exported to the cytoplasm where it can then activate the JNK signaling pathway [43] (Figure 3). The discovery that JNK itself is a direct SUMO conjugation target suggests that this signaling pathway may be regulated at multiple levels by SUMOylation [12].

## 6. Metamorphosis

A role for SUMOylation in triggering metamorphosis was first suggested by the observation that *Ubc9* mutant larvae exhibit prolonged larval life, but die before pupariation [44]. Consistent with this observation, RNAi-mediated depletion of SUMO from the ecdysone (E)-producing prothoracic gland (PG) resulted in developmental arrest at the third instar larval stage (L3). These larvae failed to pupariate but continued to grow in size (achieving twice their normal weight) and survived for an additional three weeks [45]. E is a precursor to 20-hydroxyecdysone (20E), a hormone that is a crucial regulator of metamorphosis. SUMO depleted larvae had reduced total ecdysteroid levels, suggesting that the L3 arrest phenotype was due to an inability to produce 20E. Consistent with this idea, feeding the SUMO knock-down larvae media containing 20E prevented the L3 developmental arrest and allowed pupariation, although the pupae failed to develop into adults [45].

The reduced levels of 20E caused by loss of SUMO in the PG can be explained by changes in the localization and expression levels of steroidogenic factors. The PG-expressed genes *phantom* (*phm*), *disembodied* (*dib*) and *shadow* (*sad*) encode for cytochrome p450 enzymes that are responsible for converting cholesterol to 20E [46]. SUMO depletion in the PG had no effect on Phm. However, Dib protein was present at a lower level in the mitochondria while Sad (normally nuclear and cytoplasmic) accumulated in the cytoplasm and was excluded from the nucleus. Multiple transcription factors that regulate expression of the ecdysteroid biosynthesis enzymes also displayed aberrant localization and expression levels in PG cells with reduced SUMO or Ubc9.

SUMO RNAi produced morphological changes in the plasma membrane and nuclei of PG cells but did not result in obvious structural defects in the ER or mitochondria (the primary sites of E biosynthesis from cholesterol). A thickening of the nuclear lamina beneath the inner nuclear membrane was observed, however this did not appear to affect the distribution or function of the nuclear pores as a global effect on nucleo-cytoplasmic transport was not apparent. In wild-type PG cells, the plasma membrane forms many invaginations that extend deep within the cell and the formation of these intracellular channels is believed to facilitate the process of lipid uptake and ecdysteroid secretion. In SUMO depleted PG cells the number and size of these intracellular channels was significantly reduced and the accumulation of sterol-containing lipid droplets in the cytoplasm was inhibited. These findings suggest that the impaired metamorphosis phenotype observed in SUMO knock-down larvae results from a reduction in cholesterol uptake in the PG cells that contributes to an inability to produce the ecdysteroid levels required for the developmental transition from the larval to the pupal stage [45]. 

## 7. Neurogenesis

A number of SUMO conjugation targets have essential roles in the development of both the central and peripheral nervous system. Tramtrack69 (Ttk69) is a transcriptional repressor that antagonizes neuronal fate determination in the peripheral nervous system [47]. Sumoylated Ttk69 binds to its target DNA sequence with an affinity equal to that of the unmodified protein and Ttk69 colocalizes with SUMO on polytene chromosomes. While the functional effects of SUMOylation on Ttk69-mediated repression remain unknown, the high levels of SUMO present in sensory bristle cells suggest a possible role for SUMOylation during sense organ differentiation [26].

The activity of SoxNeuro, a member of the SRY high mobility group (HMG) box (Sox) family with roles in central nervous system (CNS) development also appears to be regulated by SUMOylation. Preventing SUMOylation of SoxNeuro (by mutating the single SUMO acceptor lysine to an arginine or through expression of a dominant-negative form of Ubc9) resulted in increased SoxNeuro-mediated transcriptional activity. While embryonic overexpression of wild-type SoxNeuro had no detectable effect on CNS development, overexpression of the SoxNeuro SUMOylation deficient lysine to arginine mutant resulted in significant CNS defects including missing or reduced longitudinal axon tracts, absent or fused neural commissures, and an aberrant axonal fasciculation pattern. These results suggest that repression of SoxNeuro transcriptional activity by SUMOylation is necessary for the proper development of the embryonic CNS [48]. 

The zinc finger transcription factor Senseless (Sens), which has a role in peripheral nervous system development, also appears to be directly modulated by SUMOylation [49]. Sens is a member of the GPS (Gri1/Pag-3/Senseless) family of proteins and plays an essential role in maturation of sense organ precursor (SOPs) by synergizing with proneural basic-helix-loop-helix (bHLH) transcription factors as they autoregulate the genes that encode them. Sens is a direct target for SUMOylation and mutagenesis of its SUMO-acceptor lysine reduces synergy between Sens and the proneural transcription factors both in cell culture and in vivo. This suggests that SUMOylation of Sens promotes its synergistic interaction with proneural proteins, to regulate a critical step in the maturation of the SOPs [49]. 

## 8. Innate Immunity

The innate immune response in *Drosophila* consists of a humoral component (the expression of anti-microbial peptides in the fat body) and a cellular component (the production of hemocytes capable of recognizing and neutralizing foreign material through phagocytosis and/or encapsulation). Multiple studies have shown that the SUMO conjugation pathway acts as a critical negative effector of Toll/Rel family-mediated regulation of anti-microbial peptide expression and hematopoiesis. Similar to phenotypes observed with Toll gain-of-function alleles, *Ubc9* mutants display “melanotic tumors”, an overproliferation of hemocytes, and they exhibit constitutive expression of the anti-microbial peptides Drosomycin and Cecropin in the absence of immune challenge. These defects in hematopoietic proliferation and anti-microbial peptide expression are suppressed by mutations in the Rel family genes *dorsal* and *dif* [44]. In *Ubc9* mutant hemocytes and fat body cells, Dorsal and Dif accumulate in the nucleus in a manner similar to that observed during activation of the Toll signaling pathway [50,51]. In the absence of Toll signaling, Dorsal is retained in the cytoplasm through an interaction with the IκB family protein Cactus. Cactus protein levels are significantly reduced in *Ubc9* mutant fat body cells and a gain-of-function allele of *Cactus* (resistant to Toll signal-dependent degradation) suppresses “tumorigenesis”, overproduction of hemocytes, and constitutive expression of Drosomycin caused by loss of Ubc9 activity [51]. These findings are consistent with the mammalian model of inhibition of the Rel family factor NFκB by SUMO, in which SUMOylation of IκB protects it from ubiquitin-mediated degradation [52]. While SUMOylation of Cactus has not been demonstrated, Ubc9 does physically associate with both Cactus and Dorsal [31].

While the above findings suggest that SUMO downregulates the innate immune response under some circumstances, SUMO may also stimulate Rel family protein activity and therefore the immune response in other contexts. For example, mutations in Ubc9 or SUMO interfere with antimicrobial peptide production in first instar larvae in response to treatment with the bacterial cell wall component lipopolysaccharide [30]. This is consistent with the observation that SUMOylation of the Rel family protein Dorsal makes it a more potent transcriptional activator in S2 cells [30,31].

## 9. SUMO as a Regulator of Groucho Function

An additional SUMOylation target that has not been discussed above and that may link SUMO’s roles in embryonic patterning, wing morphogenesis, and neurogenesis is Groucho (Gro), a factor that has broad roles in all these processes. This factor is broadly distributed in the embryo and imaginal discs. It is a corepressor, meaning that, while it is required for transcriptional repression, it does not bind to DNA directly, but rather is recruited to DNA by DNA-binding repressor proteins. It is required for the function of many of the repressors that control development [53].

**Figure 4:**
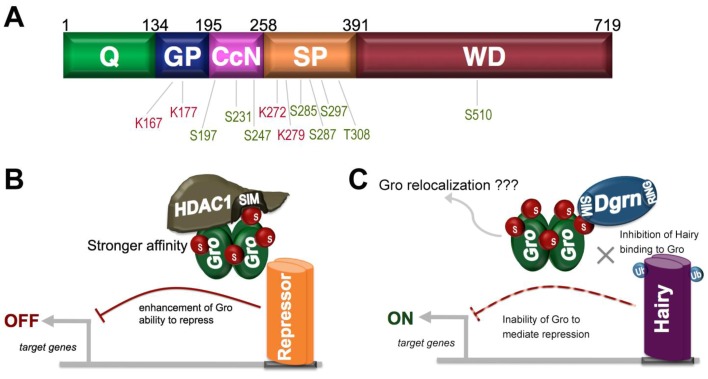
The role of SUMOylation in Groucho (Gro)-mediated repression. (**A**) Gro consists of five domains (the Q, GP, CcN, SP, and WD-repeat domains). Known SUMO acceptor residues are shown in red, while known phospho-acceptor residues are shown in green; (**B**) Proposed mechanism by which SUMOylation of Gro enhances its ability to repress by increasing its affinity for a SUMO interaction motif (SIM) in HDAC1; (**C**) Proposed mechanism by which SUMOylation of Gro inhibits Gro-mediated repression through an inhibitory interaction between sumoylated Gro and a SIM in the SUMO-targeted ubiquitin ligase Dgrn. This interaction may lead to relocalization of Gro to a cellular compartment where it is inactive. Dgrn may also direct ubiquitylation of the Gro-dependent repressor Hairy, inhibiting Hairy’s ability to interact with Gro, thus further relieving the repression of a subset of Gro targets.

Gro contains highly conversed regions at its N and C-termini (the Q and WD-repeat domains, respectively) that are essential for function and a poorly conserved central region (consisting of GP, CcN, and SP domains) [54]. While these central domains appear to be disordered, they nonetheless play critical positive and negative roles in repression–the GP and CcN domains are required for repression, while the SP domain dampens Gro function, and therefore its deletion results in Gro hyperactivity [55]. The disorder in this region could allow it to adapt to and bind multiple targets. The resulting low affinity, but high specificity interactions could be readily modulated by posttranslational modification of this region making these central domains into prime candidates for regulation by such modifications [53,55]. In accord with this idea, this region is known to be targeted for phosphorylation by a variety of protein kinases and is also the target of SUMOylation [53] (Figure 4).

Four potential SUMOylation sites have been identified in Gro, two in the positively acting GP domain and two in the negatively acting SP domain, suggesting that SUMOylation could have both positive and negative roles in controlling Gro function [56]. In support of the possibility that SUMO upregulates Gro, mutagenesis of the SUMO acceptor sites in Gro results in decreased Gro-mediated repression in mammalian cultured cells. Furthermore, Ubc9 knockdown appears to compromise repression by wild-type Gro, but not by the quadruple SUMO acceptor site mutant [56].

The ability of SUMO to enhance Gro function may result from the ability of SUMO to stabilize an interaction between Gro and the histone deacetylase HDAC1, which a number of studies have shown to be required for repression by Gro family proteins. HDAC1 contains a conserved SUMO-interaction motif (SIM). These motifs, which consist of a hydrophobic stretch and a nearby charged region, mediate non-covalent interactions with SUMO and are found in a number of SUMO-interacting proteins. In co-immunoprecipitation experiments, a SUMO-Gro fusion protein was found to bind wild-type HDAC1 more efficiently than did the Gro protein not fused to SUMO, while an HDAC1 mutant lacking the SIM did not [56]. These analyses suggest that Gro SUMOylation enhances its corepressor activity due to efficient recruitment of HDAC1 through its SIM domain (Figure 4B). These findings are consistent with the observation that two of the SUMO acceptor sites in Gro are in the GP domain, which has a major role in HDAC1 recruitment. However, it should be noted that it has not been determined if the SUMO-acceptor sites in the GP domain are specifically required for the enhancement of HDAC1 binding.

While the above findings suggest that SUMO regulates Gro function positively, another study suggests that SUMO may antagonize Gro function, which could be in accord with the presence of SUMOylation sites in the negatively acting SP domain. In this study, Gro was found to be a target of regulation by the SUMO-targeted ubiquitin ligase (STUbL) Degringolade (Dgrn) [57]. STUbLs are RING domain ubiquitin ligases that contain SIMs through which they recognize sumoylated proteins [58]. Tissue culture experiments suggest that Dgrn and Gro work antagonistically and that this antagonism requires a functional SUMO pathway [57]. For example, repression by Gro in S2 cells of a reporter under the control of a pro-neural promoter is blocked by wild-type Dgrn, but not by an inactive form of Dgrn containing a mutated RING domain [57]. Furthermore, the effect of Dgrn on Gro-mediated repression is reduced when SUMO levels are reduced. Evidence that the antagonistic interaction between Gro and Dgrn is functional in vivo is provided by the ability of Dgrn, but not the Dgrn SIM mutant, to suppress the small eye phenotype that results from overexpression of Gro in the eye disc. Similarly, Dgrn prevents the loss of wing bristles that results from Gro overexpression in the developing wing [57].

As a major role of ubiquitin is to target proteins for destruction by the proteasome, we might expect the antagonistic relationship between the ubiquitin ligase Dgrn and Gro to be the result of ubiquitin-dependent proteoloysis of Gro. Surprisingly, however, it does not appear that Gro levels are reduced in the presence of Dgrn, nor is there any evidence that Gro is ubiquitylated by Dgrn. Rather, it appears that, in the presence of Dgrn, Gro may become sequestered to some cellular compartment where it is inactive (Figure 4C), as suggested by the observation that extraction of sumoylated Gro from Dgrn-containing cells requires harsher conditions that does extraction of sumoylated Gro from Dgrn-deficient cells. 

## 10. A Potential Role for p53 SUMOylation in Apoptosis

Programmed cell death, apoptosis, plays widespread roles in development by eliminating excess cells and thus helping to sculpt structures during morphogenesis. As already mentioned above, SUMO may regulate apoptosis in response to JNK signaling by modulating Hipk subcellular localization. In addition, SUMO may regulate apoptosis via effects on the activity of the tumor suppressor protein p53. This DNA binding transcription factor is critical for triggering the expression of apoptotic genes, and the *Drosophila* p53 orthologue (Dmp53) is known to activate the expression of pro-apoptotic genes such as Reaper, Grim, and Hid. In the *Drosophila* eye, apoptosis is a normal part of development, serving to remove excess cells during pupal eye development, and can also be induced by events such as DNA damage that interfere with normal cell cycle progression.

In mammals, SUMOylation has been implicated as a regulator of p53 subcellular localization, nucleocytoplasmic translocation, and intermolecular interactions including DNA-binding [59,60]. However, the biological relevance of these functions is controversial [61,62]. Like mammalian p53, Dmp53 appears to be a target of SUMOylation [63,64,65,66]. However, unlike mammalian p53, which contains a single SUMO acceptor lysine, Dmp53 contains two such residues located at opposite ends of the protein, neither of which seems to correspond to the SUMO acceptor lysine in the mammalian protein. Reporter assays in *Drosophila* S2 cells suggest that these two SUMO acceptor residues are required for full Dmp53 transcriptional activity [63]. The fact that either SUMO acceptor site alone is sufficient for activity, suggests that SUMO does not modify p53 activity through a direct effect on one of its biochemical functions (e.g., DNA binding, oligomerization), but rather that SUMO just needs to be present at the site of Dmp53 action, perhaps to recruit coregulatory factors [63,66].

In support of the notion that Dmp53 SUMOylation modulates the apoptotic response in development, it was found that mutagenesis of the two SUMO acceptor lysines attenuated the apoptotic response that results from ionizing radiation-induced DNA damage in larval eye discs. [63]. This is consistent with studies conducted in mammalian cultured cells and in mice in which mutations in p53 SUMO acceptor sites were found to cause defects in the DNA damage response [67,68]. In sum, these results suggest that Dmp53 SUMOylation is required for induction of apoptosis following DNA damage. However, genetic evidence that SUMO pathway components contribute to the apoptotic response is lacking. 

## 11. Conclusions

SUMO is a reversible regulatory switch that can be used to control many (perhaps most) developmental processes [69]. This regulation is often complex and often targets multiple components of a pathway, sometimes in contrasting ways. In a sense, attempting to determine the role of SUMOylation in development is like trying to formulate a coherent picture regarding the role of any common protein modification (e.g., phosphorylation, acetylation, glycosylation, *etc*.) in development. The pleiotropic roles of SUMO together with the redundancy associated with the existence of multiple SUMOylation targets in any given pathway and multiple SUMO acceptor sites in any given target have made this analysis a challenge. To overcome the challenge of interpreting data produced from experiments in which SUMOylation has been globally perturbed, SUMO acceptor site mutations within the substrate have been utilized. However, this approach can be complicated by the possibility that these sites could serve as acceptors for other posttranslational modifications (e.g., acetylation and methylation). An experimental approach that combines SUMO acceptor site mutants, SUMO-substrate fusions, and overexpression/knockdown of pathway components is perhaps the best way to gain a clear picture of the effects of substrate-specific SUMO modification.

An additional complicating factor in studies of the biological roles of SUMOylation is the so-called “SUMO enigma” [60]. In particular, it is often found that only a small proportion of the population of any given substrate needs to be sumoylated to see robust effects on substrate targeting or activity. This suggests that cyclic conjugation and deconjugation may leave behind an unmodified protein that nonetheless retains a memory of having been sumoylated. For example, perhaps SUMOylation leads to the formation of macromolecular complexes, which then, because of some kind of “hysteresis”, hold together even after deconjugation. Alternatively, perhaps cyclic conjugation and deconjugation serves to take target proteins through multiple functional states that are needed in succession for pathway activity.

Given the above complexities, a facile genetic model, such as *Drosophila*, has proved invaluable in efforts to decipher the roles of SUMO in development. Using such a model, it has been possible to combine biochemical and cell culture studies with genetic studies targeting the genes encoding the proteins in the SUMOylation pathway, and with genetic studies targeting the SUMO acceptor sites in the substrate proteins. In this way, it has been possible to build up compelling evidence for functional links between numerous SUMOylation substrates and a variety of developmental pathways.

## References

[1] Kumar D., Misra J.R., Kumar A., Chugh J., Sharma S., Hosur R.V. (2009). NMR-derived solution structure of SUMO from Drosophila melanogaster (dSmt3). Proteins.

[2] Bohren K.M., Nadkarni V., Song J.H., Gabbay K.H., Owerbach D. (2004). A M55V polymorphism in a novel SUMO gene (SUMO-4) differentially activates heat shock transcription factors and is associated with susceptibility to type I diabetes mellitus. J. Biol. Chem..

[3] Saitoh H., Hinchey J. (2000). Functional heterogeneity of small ubiquitin-related protein modifiers SUMO-1 *versus* SUMO-2/3. J. Biol. Chem..

[4] Huang H.W., Tsoi S.C., Sun Y.H., Li S.S. (1998). Identification and characterization of the SMT3 cDNA and gene encoding ubiquitin-like protein from Drosophila melanogaster. Biochem. Mol. Biol. Int..

[5] Smith M., Bhaskar V., Fernandez J., Courey A.J. (2004). Drosophila Ulp1, a nuclear pore-associated SUMO protease, prevents accumulation of cytoplasmic SUMO conjugate. J. Biol. Chem..

[6] Long X., Griffith L.C. (2000). Identification and characterization of a SUMO-1 conjugation system that modifies neuronal calcium/calmodulin-dependent protein kinase II in Drosophila melanogaster. J. Biol. Chem..

[7] Rodriguez M.S., Dargemont C., Hay R.T. (2001). SUMO-1 conjugation *in vivo* requires both a consensus modification motif and nuclear targeting. J. Biol. Chem..

[8] Ulrich H.D. (2008). The fast-growing business of SUMO chains. Mol. Cell.

[9] Stielow B., Sapetschnig A., Kruger I., Kunert N., Brehm A., Boutros M., Suske G. (2008). Identification of SUMO-dependent chromatin-associated transcriptional repression components by a genome-wide RNAi screen. Mol. Cell.

[10] Betz A., Lampen N., Martinek S., Young M.W., Darnell J.E. (2001). A Drosophila PIAS homologue negatively regulates stat92E. Proc. Natl. Acad. Sci. USA.

[11] Apionishev S., Malhotra D., Raghavachari S., Tanda S., Rasooly R.S. (2001). The Drosophila UBC9 homologue lesswright mediates the disjunction of homologues in meiosis I. Genes Cells.

[12] Nie M., Xie Y., Loo J.A., Courey A.J. (2009). Genetic and proteomic evidence for roles of Drosophila SUMO in cell cycle control, Ras signaling, and early pattern formation. PloS One.

[13] Capelson M., Corces V.G. (2006). SUMO conjugation attenuates the activity of the gypsy chromatin insulator. EMBO J..

[14] Capelson M., Corces V.G. (2005). The ubiquitin ligase dTopors directs the nuclear organization of a chromatin insulator. Mol. Cell.

[15] Matsui M., Sharma K.C., Cooke C., Wakimoto B.T., Rasool M., Hayworth M., Hylton C.A., Tomkiel J.E. (2011). Nuclear structure and chromosome segregation in Drosophila male meiosis depend on the ubiquitin ligase dTopors. Genetics.

[16] Golovnin A., Volkov I., Georgiev P. (2012). SUMO conjugation is required for the assembly of Drosophila Su(Hw) and Mod(mdg4) into insulator bodies that facilitate insulator complex formation. J. Cell Sci..

[17] Schotta G., Ebert A., Krauss V., Fischer A., Hoffmann J., Rea S., Jenuwein T., Dorn R., Reuter G. (2002). Central role of Drosophila SU(VAR)3-9 in histone H3-K9 methylation and heterochromatic gene silencing. EMBO J..

[18] Hari K.L., Cook K.R., Karpen G.H. (2001). The Drosophila Su(var)2-10 locus regulates chromosome structure and function and encodes a member of the PIAS protein family. Genes Dev..

[19] Reo E., Seum C., Spierer P., Bontron S. (2010). Sumoylation of Drosophila SU(VAR)3-7 is required for its heterochromatic function. Nucleic Acids Res..

[20] Simon J.A., Kingston R.E. (2009). Mechanisms of polycomb gene silencing: Knowns and unknowns. Nat. Rev. Mol. Cell Biol..

[21] Smith M., Mallin D.R., Simon J.A., Courey A.J. (2011). Small ubiquitin-like modifier (SUMO) conjugation impedes transcriptional silencing by the polycomb group repressor Sex Comb on Midleg. J. Biol. Chem..

[22] Lewis E.B. (1998). The bithorax complex: The first fifty years. Int. J. Dev. Biol..

[23] Kagey M.H., Melhuish T.A., Wotton D. (2003). The polycomb protein Pc2 is a SUMO E3. Cell.

[24] Schnorr J.D., Holdcraft R., Chevalier B., Berg C.A. (2001). Ras1 interacts with multiple new signaling and cytoskeletal loci in Drosophila eggshell patterning and morphogenesis. Genetics.

[25] Schweitzer R., Shilo B.Z. (1997). A thousand and one roles for the Drosophila EGF receptor. Trends Genet..

[26] Lehembre F., Badenhorst P., Muller S., Travers A., Schweisguth F., Dejean A. (2000). Covalent modification of the transcriptional repressor tramtrack by the ubiquitin-related protein Smt3 in Drosophila flies. Mol. Cell Biol..

[27] Hashiyama K., Shigenobu S., Kobayashi S. (2009). Expression of genes involved in sumoylation in the Drosophila germline. Gene Expr. Patterns.

[28] Epps J.L., Tanda S. (1998). The Drosophila semushi mutation blocks nuclear import of bicoid during embryogenesis. Curr. Biol..

[29] Liu J., Ma J. (2012). Drosophila Bicoid is a substrate of sumoylation and its activator function is subject to inhibition by this post-translational modification. FEBS Lett..

[30] Bhaskar V., Smith M., Courey A.J. (2002). Conjugation of Smt3 to dorsal may potentiate the Drosophila immune response. Mol. Cell Biol..

[31] Bhaskar V., Valentine S.A., Courey A.J. (2000). A functional interaction between dorsal and components of the Smt3 conjugation machinery. J. Biol. Chem..

[32] Miles W.O., Jaffray E., Campbell S.G., Takeda S., Bayston L.J., Basu S.P., Li M., Raftery L.A., Ashe M.P., Hay R.T. (2008). Medea SUMOylation restricts the signaling range of the Dpp morphogen in the Drosophila embryo. Genes Dev..

[33] Affolter M., Marty T., Vigano M.A., Jazwinska A. (2001). Nuclear interpretation of Dpp signaling in Drosophila. EMBO J..

[34] Gao S., Steffen J., Laughon A. (2005). Dpp-responsive silencers are bound by a trimeric Mad-Medea complex. J. Biol. Chem..

[35] Long J., Wang G., He D., Liu F. (2004). Repression of Smad4 transcriptional activity by SUMO modification. Biochem. J..

[36] Takanaka Y., Courey A.J. (2005). SUMO enhances vestigial function during wing morphogenesis. Mech. Dev..

[37] De Celis J.F., Barrio R. (2009). Regulation and function of Spalt proteins during animal development. Int. J. Dev. Biol..

[38] Netzer C., Bohlander S.K., Rieger L., Muller S., Kohlhase J. (2002). Interaction of the developmental regulator SALL1 with UBE2I and SUMO-1. Biochem. Biophys. Res. Commun..

[39] Sanchez J., Talamillo A., Lopitz-Otsoa F., Perez C., Hjerpe R., Sutherland J.D., Herboso L., Rodriguez M.S., Barrio R. (2010). Sumoylation modulates the activity of Spalt-like proteins during wing development in Drosophila. J. Biol. Chem..

[40] Sanchez J., Talamillo A., Gonzalez M., Sanchez-Pulido L., Jimenez S., Pirone L., Sutherland J.D., Barrio R. (2011). Drosophila Sal and Salr are transcriptional repressors. Biochem. J..

[41] Hofmann T.G., Stollberg N., Schmitz M.L., Will H. (2003). HIPK2 regulates transforming growth factor-beta-induced c-Jun NH(2)-terminal kinase activation and apoptosis in human hepatoma cells. Cancer Res..

[42] Lan H.C., Li H.J., Lin G., Lai P.Y., Chung B.C. (2007). Cyclic AMP stimulates SF-1-dependent CYP11A1 expression through homeodomain-interacting protein kinase 3-mediated Jun N-terminal kinase and c-Jun phosphorylation. Mol. Cell Biol..

[43] Huang H., Du G., Chen H., Liang X., Li C., Zhu N., Xue L., Ma J., Jiao R. (2011). Drosophila Smt3 negatively regulates JNK signaling through sequestering Hipk in the nucleus. Development.

[44] Chiu H., Ring B.C., Sorrentino R.P., Kalamarz M., Garza D., Govind S. (2005). dUbc9 negatively regulates the Toll-NF-kappa B pathways in larval hematopoiesis and drosomycin activation in Drosophila. Dev. Biol..

[45] Talamillo A., Sanchez J., Cantera R., Perez C., Martin D., Caminero E., Barrio R. (2008). Smt3 is required for Drosophila melanogaster metamorphosis. Development.

[46] Warren J.T., Petryk A., Marques G., Parvy J.P., Shinoda T., Itoyama K., Kobayashi J., Jarcho M., Li Y., O’Connor M.B. (2004). Phantom encodes the 25-hydroxylase of Drosophila melanogaster and Bombyx mori: A P450 enzyme critical in ecdysone biosynthesis. Insect Biochem. Mol. Biol..

[47] Guo M., Jan L.Y., Jan Y.N. (1996). Control of daughter cell fates during asymmetric division: Interaction of Numb and Notch. Neuron.

[48] Savare J., Bonneaud N., Girard F. (2005). SUMO represses transcriptional activity of the Drosophila SoxNeuro and human Sox3 central nervous system-specific transcription factors. Mol. Biol. Cell.

[49] Powell L.M., Chen A., Huang Y.C., Wang P.Y., Kemp S.E., Jarman A.P.  (2012). The SUMO pathway promotes bHLH proneural factor activity via a direct effect on the Zn finger protein, Senseless. Mol. Cell Biol..

[50] Huang L., Ohsako S., Tanda S. (2005). The lesswright mutation activates Rel-related proteins, leading to overproduction of larval hemocytes in Drosophila melanogaster. Dev. Biol..

[51] Paddibhatla I., Lee M.J., Kalamarz M.E., Ferrarese R., Govind S. (2010). Role for sumoylation in systemic inflammation and immune homeostasis in Drosophila larvae. PLoS Pathog..

[52] Desterro J.M., Rodriguez M.S., Hay R.T. (1998). SUMO-1 modification of IkappaBalpha inhibits NF-kappaB activation. Mol. Cell.

[53] Turki-Judeh W., Courey A.J. (2012). Groucho a corepressor with instructive roles in development. Curr. Top. Dev. Biol..

[54] Stifani S., Blaumueller C.M., Redhead N.J., Hill R.E., Artavanis-Tsakonas S. (1992). Human homologs of a Drosophila Enhancer of split gene product define a novel family of nuclear proteins. Nat. Genet..

[55] Turki-Judeh W., Courey A.J. (2012). The Unconserved Groucho Central Region is essential for Viability and Modulates Target Gene Specificity. PloS One.

[56] Ahn J.W., Lee Y.A., Ahn J.H., Choi C.Y. (2009). Covalent conjugation of Groucho with SUMO-1 modulates its corepressor activity. Biochem. Biophys. Res. Commun..

[57] Abed M., Barry K.C., Kenyagin D., Koltun B., Phippen T.M., Delrow J.J., Parkhurst S.M., Orian A. (2011). Degringolade, a SUMO-targeted ubiquitin ligase, inhibits Hairy/Groucho-mediated repression. EMBO J..

[58] Abed M., Bitman-Lotan E., Orian A.  (2011). A fly view of a SUMO-targeted ubiquitin ligase. Fly (Austin).

[59] Wu S.Y., Chiang C.M. (2009). p53 sumoylation: Mechanistic insights from reconstitution studies. Epigenetics Off. J. DNA Methylation Soc..

[60] Hay R.T. (2005). SUMO: A history of modification. Mol. Cell.

[61] Melchior F., Hengst L. (2002). SUMO-1 and p53. Cell Cycle.

[62] Muller S., Ledl A., Schmidt D. (2004). SUMO: A regulator of gene expression and genome integrity. Oncogene.

[63] Mauri F., McNamee L.M., Lunardi A., Chiacchiera F., Del Sal G., Brodsky M.H., Collavin L. (2008). Modification of Drosophila p53 by SUMO modulates its transactivation and pro-apoptotic functions. J. Biol. Chem..

[64] Formstecher E., Aresta S., Collura V., Hamburger A., Meil A., Trehin A., Reverdy C., Betin V., Maire S., Brun C. (2005). Protein interaction mapping: A Drosophila case study. Genome Res..

[65] Stanyon C.A., Liu G., Mangiola B.A., Patel N., Giot L., Kuang B., Zhang H., Zhong J., Finley R.L. (2004). A *drosophila* protein-interaction map centered on cell-cycle regulators. Genome Biol..

[66] Pardi N., Vamos E., Ujfaludi Z., Komonyi O., Bodai L., Boros I.M. (2011). *In vivo* effects of abolishing the single canonical sumoylation site in the C-terminal region of Drosophila p53. Acta Biol. Hung..

[67] Muller S., Berger M., Lehembre F., Seeler J.S., Haupt Y., Dejean A. (2000). c-Jun and p53 activity is modulated by SUMO-1 modification. J. Biol. Chem..

[68] Feng L., Lin T., Uranishi H., Gu W., Xu Y. (2005). Functional analysis of the roles of posttranslational modifications at the p53 C terminus in regulating p53 stability and activity. Mol. Cell Biol..

[69] Lomeli H., Vazquez M. (2011). Emerging roles of the SUMO pathway in development. Cell Mol. Life Sci..

